# Mechanical characteristics analysis of high dimensional vibration isolation systems based on high-static-low-dynamic stiffness technology

**DOI:** 10.1038/s41598-024-58469-x

**Published:** 2024-04-08

**Authors:** Bu-yun Li, Chang-geng Shuai, Jian-guo Ma

**Affiliations:** 1 Institute of Noise & Vibration, Naval University of Engineering, Wuhan, 430030 People’s Republic of China; 2 National Key Laboratory on Ship Vibration & Noise, Wuhan, 430030 People’s Republic of China

**Keywords:** High-static-low-dynamic stiffness, Quasi-zero stiffness, High dimensional nonlinear system, Floating raft vibration isolation system, Swing stability, Engineering, Mechanical engineering

## Abstract

Large floating raft vibration isolation systems (FRVISs) based on high-static-low-dynamic stiffness (HSLDS) technology offer excellent low frequency vibration isolation performance with broad application prospects. However, the design process for these complex high-dimensional coupled nonlinear systems remains poorly developed, particularly when applied for ocean-going vessels that experience rolling and pitching motions. The present work addresses this issue by establishing a six-degree-of-freedom HSLDS vibration isolation model for FRVISs composed of eight isolators, and the model is applied to fully analyze the swing stability and multidimensional vibration isolation performance of these systems. The influence of nonlinearity on the mechanical properties of the vibration isolators is analyzed more clearly by assuming that each vibration isolator realizes nonlinear HSLDS characteristics in the *z* direction and linear characteristics in the *x* and *y* directions. The results demonstrate that the swing displacement responses of the system are greatly reduced under weak nonlinearity, which reflects the high static stiffness and high static stability characteristics of an HSLDS system. The multidimensional vibration isolation performance of the system is evaluated according to the impacts of nonlinearity, the installation height *H*_*z*_ of the isolators, and the relative position *D*_*r*_ of the two middle isolators. The results of analysis demonstrate that applying a value of *H*_*z*_ = 0 produces the best vibration isolation performance overall under strong nonlinearity by avoiding unnecessary secondary peaks in the force transmission rate under harmonic mechanical excitation and ensuring a maximum high-frequency vibration isolation effect. However, applying a weak nonlinearity is better than a strong nonlinearity if *H*_*z*_ is not zero. In contrast, *D*_*r*_ has little effect on the vibration isolation effect of the raft in the *x*, *y*, and *z* directions. Therefore, an equidistant installation with *D*_*r*_ = 0.5 would be considered ideal from the standpoint of installation stability.

## Introduction

Floating raft vibration isolation systems (FRVISs) represent a state-of-the-art technology for minimizing the mechanical vibrations of ocean going vessels with a range of goals, such as for enhancing their acoustic stealth performance. Of particular importance in this regard is to maximize the low-frequency vibration isolation performance of these systems^1^. From a theoretical perspective, the low-frequency vibration isolation performance of these systems increases as their natural frequency decreases^2^. The primary means of reducing the natural frequency of an FRVIS is to reduce the stiffness of the vibration isolators applied therein. However, this strategy suffers from at least two problems. First, the lowest natural frequency of an isolator is limited by the physical properties, and cannot be reduced indefinitely. Second, system stability is increasingly compromised under decreasing stiffness because this also increases the deformation experienced by the system under the rolling and pitching conditions of the vessel.

These issues have been addressed in recent years by the development of high-static-low-dynamic stiffness (HSLDS) vibration isolation structures, which can reduce dynamic stiffness while ensuring static stability^3,4^. Ideally, the dynamic stiffness of this type of vibration isolation structure can approach a value of zero, and therefore represents quasi-zero stiffness (QZS) performance^5^. At present, a number of HSLDS vibration isolator structures have been designed, such as inclined spring structures^6–10^, cam-roller structures^11–15^, pneumatic structures^16–18^, magnetic structures^19–22^, and structures inspired by biological organisms^23–26^. Moreover, Li and Xu^27,28^ were the first to design an FRVIS using QZS isolators, and the vibration isolation performance of the resulting system was analyzed. However, while the low-frequency vibration isolation performance of the system was demonstrated to be substantially improved via the use of QZS isolators, the model established was a little simple, and some factors affecting the vibration isolation effect of the system were not considered, such as the number of isolators, which can be very many in an FRVIS, and their installation positions. In addition, the influence of different excitation conditions and the nonlinearities of the isolators on the vibration isolation performance of FRVISs were also not considered. For example, the vibration isolation characteristics of the system were evaluated under non-eccentric excitations when the isolators realized QZS condition. However, these characteristics are not at all certain under all possible excitation conditions with non-QZS characteristics. The past research results inspired the research of this paper. This paper further studies the situations that have not been considered before to ensure engineering practicability.

Moreover, in contrast to land-based applications, ocean-going vessels experience rolling and pitching motions during operation, which introduce more rigorous stability requirements for HSLDS-FRVISs. However, the swing stability of these complex vibration isolation systems remains poorly evaluated. Meanwhile, some scholars have analyzed the swing stability of linear systems. For example, He et al.^29^ analyzed the rolling stability of a vibration isolation system designed for a vessel propulsion system based on a single-layer linear vibration isolation model. However, the stability characteristics of such systems are quite different from those of highly nonlinear HSLDS systems. In addition, most studies focused on analyzing the dynamic stability of nonlinear systems have considered relatively simple HSLDS vibration isolator systems, including their nonlinear dynamic behaviors, such as jumping and bifurcation^30–32^. Accordingly, the design process for these complex high-dimensional coupled nonlinear systems remains poorly developed in ocean-going vessel applications. As a result, the design of efficient, safe, and stable HSLDS-FRVISs remains beyond the reach of the current state of the art.

The present work addresses this issue by establishing a six degrees-of-freedom (6-DOF) HSLDS-FRVIS model, and the model is applied to fully analyze the swing stability and multidimensional vibration isolation performance of these systems. Therefore, the current work lays a sound theoretical foundation for the subsequent design of HSLDS-FRVISs.

## Simplified HSLDS-FRVIS model

**Figure 1:**
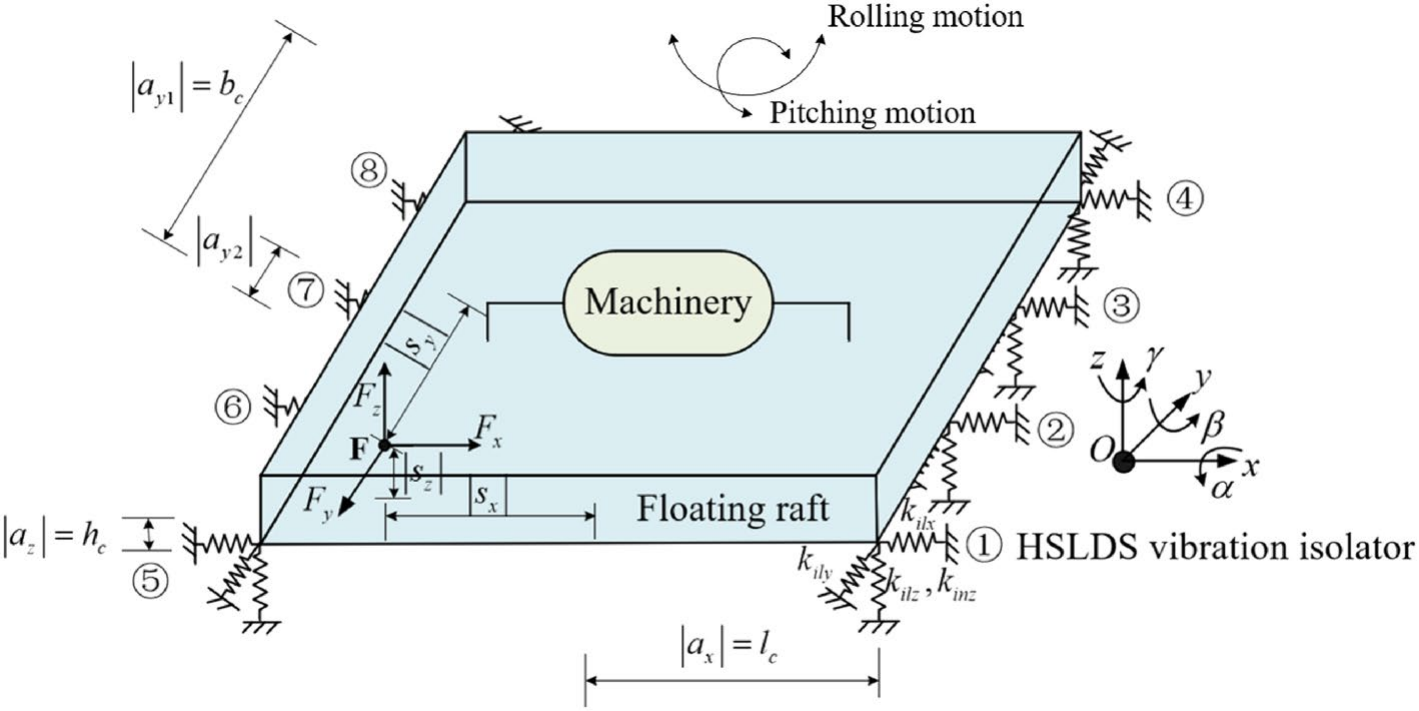
Schematic illustrating the simplified structure of a high-static-low-dynamic stiffness (HSLDS) floating raft vibration isolation system (FRVIS).

For the convenience of analysis, it is assumed that the floating raft is rigid and supported only vertically by vibration isolators. For large floating rafts, vibration isolators will be symmetrically arranged at the four corners and the middle of the raft to ensure stability. According to the actual engineering needs, 6, 8, 10 or even more vibration isolators can be symmetrically installed. For general discussion, it is assumed that the floating raft is supported by eight vibration isolators, which is also a common isolator configuration for ship vibration isolation systems. Therefore, the simplified structure of the HSLDS-FRVIS investigated in the present study is illustrated schematically in Fig. 1. As can be seen, the mechanical equipment is rigidly installed on top of the floating raft, and the floating raft is supported by eight HSLDS vibration isolators. The eight vibration isolators are numbered ①–⑧ counterclockwise from the lower right corner. The global coordinate system *OXYZ* is located at the center of gravity jointly determined by the mechanical equipment and floating raft. The coordinate system of each vibration isolator coincides with the global coordinate system. The influence of nonlinearity on the mechanical properties of the vibration isolators is analyzed more clearly by assuming that each vibration isolator realizes nonlinear HSLDS characteristics in the *z* direction and linear characteristics in the *x* and *y* directions. The other model parameters include *l*_*c*_, *b*_*c*_, and *h*_*c*_, which are one-half of the length, width, and height of the raft, respectively. In addition, installation positions *a*_*x*_, *a*_*y*1_, and *a*_*z*_ represent the coordinates of the vibration isolators in the coordinate system *OXYZ*, where $\left| {a_{x} } \right| = l_{c}$ and $\left| {a_{y1} } \right| = b_{c}$. As can be seen, the positions of vibration isolators installed at the four corners of the floating raft structure are usually determined by the length and width of the floating raft. However, the positions of the middle isolators are arbitrarily adjustable. Therefore, we define a coordinate *a*_*y*2_, which represents the position of the middle vibration isolators (i.e., isolators ②, ③, ⑥, and ⑦) relative to the center of gravity along the *y* axis. In addition, while the installation heights of the isolators are arbitrarily adjustable, we apply a standard position of $\left| {a_{z} } \right| = h_{c}$ herein unless otherwise specified. $\left| {a_{z} } \right|$ is the distance between the upper end of the isolator with rated load and the center of gravity jointly determined by the mechanical equipment and floating raft. Therefore, a standard position of $\left| {a_{z} } \right| = h_{c}$ means that the vibration isolators are installed on the bottom of the floating raft. Furthermore, the installation height of the isolators and the positions of the middle isolators are defined according to an installation height ratio $H_{z} = \left| {{{a_{zi} } \mathord{\left/ {\vphantom {{a_{zi} } {h_{c} }}} \right. \kern-0pt} {h_{c} }}} \right|$ (*i* = 1, 2, …, 8) and a distance ratio $D_{r} = \left| {{{a_{ym} } \mathord{\left/ {\vphantom {{a_{ym} } {b_{c} }}} \right. \kern-0pt} {b_{c} }}} \right|$ (*m* = 2, 3, 6, 7), respectively. The standard values of *H*_*z*_ and *D*_*r*_ applied are 1 and 0.5 respectively. Unless otherwise specified, the structural parameters applied herein are listed in Table 1. The coordinates of an applied excitation force ${\mathbf{F}}$ are denoted as $(s_{x} ,s_{y} ,s_{z} )$, where the absolute values of $s_{x}$, $s_{y}$, and $s_{z}$ respectively represent the eccentric distances of **F** in the *x*, *y*, and *z* directions. If $s_{x} = s_{y} = s_{z} = 0$, the excitation force is located at the center of gravity of the system (i.e., at *O*).

**Table 1:** Standard structural parameters applied herein for the HSLDS-FRVIS illustrated in Fig. 1.

Parameter	${{a_{x} } \mathord{\left/ {\vphantom {{a_{x} } {l_{c} }}} \right. \kern-0pt} {l_{c} }}$	${{a_{y1} } \mathord{\left/ {\vphantom {{a_{y1} } {l_{c} }}} \right. \kern-0pt} {l_{c} }}$	${{a_{y2} } \mathord{\left/ {\vphantom {{a_{y2} } {l_{c} }}} \right. \kern-0pt} {l_{c} }}$	${{a_{z} } \mathord{\left/ {\vphantom {{a_{z} } {l_{c} }}} \right. \kern-0pt} {l_{c} }}$	$H_{z}$	$D_{r}$
Value	1.0	2.4	1.2	0.07	1	0.5

The dynamic equation of the system is given as follows^33,34^. The nonlinearity includes the linear stiffness term and the cubic nonlinear stiffness term, which is a part of a typical duffing equation.

1 $$ \begin{aligned} {\mathbf{M\ddot{x}}} + \sum\limits_{i = 1}^{{n_{i} }} {{\mathbf{G}}_{i}^{T} {\mathbf{C}}_{i} } ({\mathbf{G}}_{i} {\dot{\mathbf{x}}}) + \sum\limits_{i = 1}^{{n_{i} }} {{\mathbf{G}}_{i}^{T} {\mathbf{K}}_{il} } ({\mathbf{G}}_{i} {\mathbf{x}}) \hfill \\ \quad + \sum\limits_{i = 1}^{{n_{i} }} {{\mathbf{G}}_{i}^{T} {\mathbf{K}}_{in} } (({\mathbf{G}}_{i} {\mathbf{x}}) \otimes ({\mathbf{G}}_{i} {\mathbf{x}}) \otimes ({\mathbf{G}}_{i} {\mathbf{x}})) = {\mathbf{F}} \hfill \\ \end{aligned} $$

Here, the operation $\otimes$ is defined as the multiplications of elements in the same position of different matrices. ${\mathbf{M}}$ is a matrix containing the mass *m*, the moments of inertia $I_{ii}$ (*i* = *x*, *y*, *z*), and the products of inertia $I_{ij}$ (*i*, *j* = *x*, *y*, *z*, $i \ne j$) of the machinery and floating raft, which is defined as follows.

2 $$ {\mathbf{M}} = \left[ {\begin{array}{*{20}c} m & 0 & 0 & 0 & 0 & 0 \\ 0 & m & 0 & 0 & 0 & 0 \\ 0 & 0 & m & 0 & 0 & 0 \\ 0 & 0 & 0 & {I_{xx} } & { - I_{xy} } & { - I_{xz} } \\ 0 & 0 & 0 & { - I_{yx} } & {I_{yy} } & { - I_{yz} } \\ 0 & 0 & 0 & { - I_{zx} } & { - I_{zy} } & {I_{zz} } \\ \end{array} } \right] $$

$${\mathbf{x}} = \left[ {\begin{array}{*{20}c} {x_{c} } & {y_{c} } & {z_{c} } & {\alpha_{c} } & {\beta_{c} } & {\gamma_{c} } \\ \end{array} } \right]^{{\text{T}}}$$ is the displacement vector of the center of gravity, including translation terms ([*x*_*c*_* y*_*c*_* z*_*c*_]^T^) and angle terms ([*α β γ*]^T^), *n*_*i*_ is the number of the isolators, $${\mathbf{G}}_{i} = \left[ {\begin{array}{*{20}c} {} & 0 & {a_{zi} } & { - a_{yi} } \\ {{\mathbf{E}}_{3 \times 3} } & { - a_{zi} } & 0 & {a_{xi} } \\ {} & {a_{yi} } & { - a_{xi} } & 0 \\ \end{array} } \right]$$ is the position transformation matrix from the upper end of the *i*-th isolator to *O*, where ${\mathbf{E}}_{3 \times 3}$ is a $3 \times 3$ identity matrix, and $a_{\upsilon i}$($\upsilon = x,y,z$) are the above-defined vibration isolator coordinates, ${\mathbf{C}}_{i} = {\mathbf{T}}_{i}^{{\text{T}}} diag(c_{ix} ,c_{iy} ,c_{iz} ){\mathbf{T}}_{i}$ is the linear damping matrix, where ${\mathbf{T}}_{i}$ is an identity matrix because the coordinate system of each vibration isolator coincides with the global coordinate system, ${\mathbf{K}}_{il} = {\mathbf{T}}_{i}^{{\text{T}}} diag(k_{ilx} ,k_{ily} ,k_{ilz} ){\mathbf{T}}_{i}$ is the linear stiffness matrix, and ${\mathbf{K}}_{in} = {\mathbf{T}}_{i}^{{\text{T}}} diag(0,0,k_{inz} ){\mathbf{T}}_{i}$ is the nonlinear stiffness matrix. Accordingly, we can define the excitation force vector as $${\mathbf{F}} = \left[ {\begin{array}{*{20}c} {F_{x} } & {F_{y} } & {F_{z} } & {M_{x} } & {M_{y} } & {M_{z} } \\ \end{array} } \right]^{{\text{T}}}$$.

Introducing dimensionless parameters yields the following terms:

$$\hat{x}_{c} = \frac{{x_{c} }}{{l_{c} }}$$, $$\hat{y}_{c} = \frac{{y_{c} }}{{l_{c} }}$$, $$\hat{z}_{c} = \frac{{z_{c} }}{{l_{c} }}$$, $$\hat{\alpha }_{c} = \frac{{\alpha_{c} }}{\theta }$$, $$\hat{\beta }_{c} = \frac{{\beta_{c} }}{\theta }$$, $$\hat{\gamma }_{c} = \frac{{\gamma_{c} }}{\theta }$$, $$\omega_{n} = \sqrt {\frac{{8k_{z} }}{m}}$$, $$\tau = \omega_{n} t$$, $$\Omega = \frac{\omega }{{\omega_{n} }}$$, $$\xi_{\upsilon } = \frac{{c_{i\upsilon } \omega_{n} }}{{k_{z} }}$$, $$\hat{k}_{il\upsilon } = \frac{{k_{il\upsilon } }}{{k_{z} }}$$, $$\hat{k}_{inz} = \frac{{k_{inz} l_{c}^{2} }}{{k_{z} }}$$, $$\hat{F}_{\upsilon } = \frac{{F_{\upsilon } }}{{8k_{z} l_{c} }}$$, $$\hat{M}_{\upsilon } = \frac{{M_{\upsilon } }}{{8k_{z} l_{c}^{2} }}$$, and $$\hat{a}_{\upsilon } = \frac{{a_{\upsilon } }}{{l_{c} }}$$ ($$\upsilon = x,y,z$$).

Here, $\omega$ is the excitation frequency, $\theta$ is a unit angle for rendering angle terms [*α β γ*]^T^ dimensionless, *τ* is dimensionless time, *ξ* is the dimensionless damping ratio and *k*_*z*_ is the static stiffness given by the static force divided by static displacement. It is assumed that the damping of an isolator is the same in all directions. For convenience, dimensionless parameters are still represented by original parameter variables except dimensionless time $\tau$ and damping ratio $\xi$.

These dimensionless parameters transform Eq. (1) into a dimensionless dynamic equation of the system. It is further noted that the linear and nonlinear stiffness elements can be alternatively defined as $k_{ilz} = 1 - 2\left( {\frac{{1 - \hat{l}}}{{\hat{l}}}} \right)$ and $k_{inz} = \frac{{1 - \hat{l}^{2} }}{{\hat{l}^{3} }}$^34,35^. The nonlinearity of the system can be conveniently represented by the dimensionless compression factor $\hat{l}$ which is the ratio of the compressed length to the original length for springs, because *k*_*inz*_ increases while *k*_*ilz*_ decreases with increasing $\hat{l}$. Here, the system attains maximum nonlinearity when $\hat{l} = 0.667$, and is accordingly a QZS system. In contrast, a value $\hat{l} = 1$ represents an equivalent linear system (ELS).

## Static swing stability analysis

Swing motion includes rolling and pitching motions. Under rolling motion, the force exerted on the *XOZ* plane produces only translational displacements in the *x* and *z* directions and rotational displacements $\beta$ around the *y* axis, as shown in Fig. 1. Regardless of damping, the dimensionless equations defining the rolling motion of the 3-DOF system based on the 6-DOF system described in “Simplified HSLDS-FRVIS model” section are given as follows.

3 $$ \left\{{\begin{array}{ll} {\ddot{x}_{c} - \frac{1}{4}a_{z} \beta_{c} k_{lx} + \frac{1}{4}k_{lx} x_{c} = F_{x} } \\ {\ddot{z}_{c} + \frac{1}{4}k_{lz} z_{c} + \frac{1}{8}k_{nz} \left( { - a_{x} \beta_{c} + z_{c} } \right)^{3} + \frac{1}{8}k_{nz} \left( {a_{x} \beta_{c} + z_{c} } \right)^{3} = F_{z} } \\ {\ddot{\beta }_{c} + \frac{1}{4}\beta_{c} \left( {a_{z}^{2} k_{lx} + a_{x}^{2} k_{lz} } \right) - \frac{1}{4}a_{z} k_{lx} x_{c} - \frac{1}{8}a_{x} k_{nz} \left( { - a_{x} \beta_{c} + z_{c} } \right)^{3} + \frac{1}{8}a_{x} k_{nz} \left( {a_{x} \beta_{c} + z_{c} } \right)^{3} = M_{y} } \\ \end{array} } \right. $$

Here, *k*_*lx*_,* k*_*lz*_ are the linear stiffness in the *x*, *z* directions respectively. *k*_*nz*_ is the nonlinear stiffness in the *z* direction.

Under pitching motion, the force exerted on the *YOZ* plane produces only translational displacements in the *y* and *z* directions and rotational displacements $\alpha$ around the *x* axis, as shown in Fig. 1. Regardless of damping, the dimensionless equations defining the pitching motion of the corresponding 3-DOF system are defined as follows.

4 $$ \left\{ {\begin{array}{ll} {\ddot{y}_{c} + \frac{1}{2}k_{ly} y_{c} + \frac{1}{2}k_{ly} a_{z} \alpha_{c} = F_{y} } \\ {\ddot{z}_{c} + \frac{1}{2}k_{lz} z_{c} + \frac{3}{4}\alpha_{c}^{2} \left( {a_{y1}^{2} + a_{y2}^{2} } \right)k_{nz} z_{c} + \frac{1}{2}k_{nz} z_{c}^{3} = F_{z} } \\ {\ddot{\alpha }_{c} + \frac{1}{2}a_{z}^{2} k_{ly} \alpha_{c} + \frac{1}{4}\left( {a_{y1}^{2} + a_{y2}^{2} } \right)\left( {k_{lz} + 3k_{nz} z_{c}^{2} } \right)\alpha_{c} + \frac{1}{4}\left( {a_{y1}^{4} + a_{y2}^{4} } \right)k_{nz} \alpha_{c}^{3} + \frac{1}{2}a_{z} k_{ly} y_{c} = M_{x} } \\ \end{array} } \right. $$

Here, *k*_*ly*_ is the linear stiffness in the *y* direction.

The swing stability of an FRVIS is usually determined according to its static response because the swing frequency of a ship is quite small, and swing motion can therefore be regarded as a quasi-static process^29^. In addition, we assume that all vibration isolators realize QZS characteristics in the *z* direction (i.e., $\hat{l} = 0.667$) because this enables us to obtain an analytical expression of the displacement response of the system that facilitates a clear analysis of the influence of system parameters on swing stability.

Ignoring the differential terms in Eq. (3) yields the following rolling displacement response of the FRVIS.

5 $$ \left\{ {\begin{array}{ll} {x_{c} = \frac{{4F_{x} + a_{z} \beta k_{lx} }}{{k_{lx} }}} \\ {z_{c} = \frac{{(\frac{{ - 4a_{z} F_{x} + 4a_{x} F_{z} }}{{a_{x} k_{nz} }})^{1/3} + (\frac{{4a_{z} F_{x} + 4a_{x} F_{z} }}{{a_{x} k_{nz} }})^{1/3} }}{2}} \\ {\beta_{c} = \frac{{(\frac{{4a_{z} F_{x} + 4a_{x} F_{z} }}{{a_{x} k_{nz} }})^{1/3} - (\frac{{ - 4a_{z} F_{x} + 4a_{x} F_{z} }}{{a_{x} k_{nz} }})^{1/3} }}{{2a_{x} }}} \\ \end{array} } \right. $$

In contrast, the greater complexity of the equations of pitching motion in Eq. (4) make it impossible to obtain an explicit analytical pitching displacement response for the FRVIS. Therefore, the pitching displacement response of the system is given as follows. This equation can be solved using the solve function of the MATLAB^®^ software.

6 $$ \left\{ {\begin{array}{ll} {\frac{1}{2}k_{ly} y_{c} + \frac{1}{2}k_{ly} \alpha_{c} a_{z} = F_{y} } \\ {\frac{3}{4}\alpha_{c}^{2} \left( {a_{y1}^{2} + a_{y2}^{2} } \right)k_{nz} z_{c} + \frac{1}{2}k_{nz} z_{c}^{3} = F_{z} } \\ {\frac{1}{2}a_{z}^{2} k_{ly} \alpha_{c} + \frac{3}{4}k_{nz} \left( {a_{y1}^{2} + a_{y2}^{2} } \right)z_{c}^{2} \alpha_{c} + \frac{1}{4}\left( {a_{y1}^{4} + a_{y2}^{4} } \right)k_{nz} \alpha_{c}^{3} + \frac{1}{2}a_{z} k_{ly} y_{c} = 0} \\ \end{array} } \right. $$

Similarly, the dimensionless rolling displacement responses $x_{r - l}$, $z_{r - l}$, and $\beta_{r - l}$ obtained for an ELS ($\hat{l} = 1$) with only linear stiffness elements ${\mathbf{K}}_{il} = {\mathbf{T}}_{i}^{{\text{T}}} diag(k_{z} ,k_{z} ,k_{z} ){\mathbf{T}}_{i}$ and ${\mathbf{K}}_{in} = {\mathbf{0}}$ (*i* = 1, 2, …,8) can be given as follows.

7 $$ \left\{ {\begin{array}{ll} {x_{r - l} = 4F_{x} + \frac{{4F_{x} a_{z}^{2} }}{{a_{x}^{2} }}} \\ {z_{r - l} = 4F_{z} } \\ {\beta_{r - l} = \frac{{4a_{z} F_{x} }}{{a_{x}^{2} }}} \\ \end{array} } \right. $$

The dimensionless pitching displacement responses $y_{p - l}$, $z_{p - l}$, and $\alpha_{{_{p - l} }}$ are also defined as follows.

8 $$ \left\{ {\begin{array}{ll} {y_{{_{p - l} }} = 2F_{y} + \frac{{4F_{y} a_{z}^{2} }}{{a_{y1}^{2} + a_{y2}^{2} }}} \\ {z_{{_{p - l} }} = 2F_{z} } \\ {\alpha_{{_{p - l} }} = - \frac{{4a_{z} F_{y} }}{{a_{y1}^{2} + a_{y2}^{2} }}} \\ \end{array} } \right. $$

To further simplify the analysis, we assume that the load is concentrated at the origin *O* of the global coordinate system (i.e., at the center of gravity of the system). This enables us to define the balance between the load and the support force of the vibration isolator during the rolling and pitching of the ship as being equivalent to applying the following rolling and pitching forces at *O*.

9 $$ \left\{ {\begin{array}{ll} {F_{x} = G\sin \rho } \\ {F_{z} = G(1 - \cos \rho )} \\ \end{array} } \right. $$

10 $$ \left\{ {\begin{array}{ll} {F_{y} = G\sin \phi } \\ {F_{z} = G(1 - \cos \phi )} \\ \end{array} } \right. $$

Here, $G = \frac{{G_{a} }}{{8k_{z} l_{c} }}$ is the dimensionless load of the system, where $G_{a}$ is the load, $\rho = \frac{{\rho_{a} }}{\theta }$ is the dimensionless rolling angle, where $\rho_{a}$ is the rolling angle, and $\phi = \frac{{\phi_{a} }}{\theta }$ is the dimensionless pitching angle, where $\phi_{a}$ is the pitching angle.

**Figure 2:**
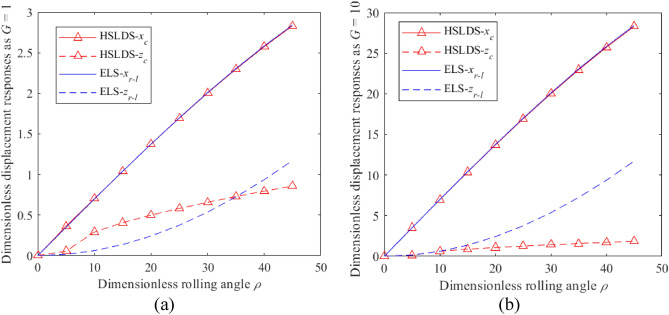
Dimensionless displacement responses obtained under rolling motion for the HSLDS-FRVIS ($\hat{l} = 0.667$) and the ELS ($\hat{l} = 1$) defined in Fig. 1 as functions of the dimensionless rolling angle *ρ* under different dimensionless loads *G*: (**a**) $G = 1$; (**b**) $G = 10$.

**Figure 3:**
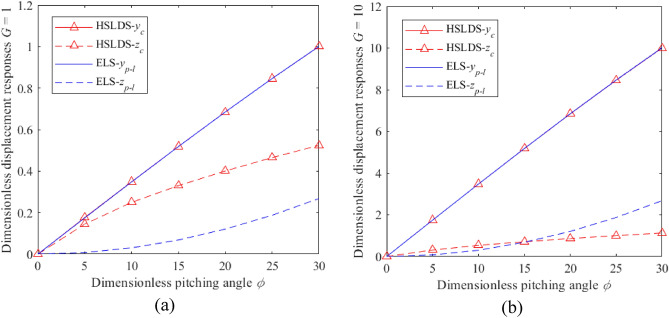
Dimensionless displacement responses obtained under pitching motion for the HSLDS-FRVIS and the equivalent linear system (ELS) as functions of the dimensionless pitching angle *φ* for different *G*: (**a**) $G = 1$; (**b**) $G = 10$.

The impacts of *ρ* and *G* on the dimensionless displacement responses of the HSLDS-FRVIS ($\hat{l} = 0.667$) and the ELS ($\hat{l} = 1$) under rolling conditions are presented in Fig. 2a and b as functions of *ρ* for *G* values of 1 and 10, respectively. Similarly, these impacts on the HSLDS-FRVIS and the ELS under pitching conditions are presented in Fig. 3a and b as functions of *φ* for *G* values of 1 and 10, respectively. It is found that the responses of the HSLDS-FRVIS and ELS are nearly equivalent in the *x* and *y* directions under the swing conditions. This demonstrates that nonlinearity in the *z* direction has little influence on the system responses in the *x* and *y* directions. Under the rolling condition, the response of the HSLDS-FRVIS in the *z* direction is generally greater than that of the ELS with increasing *ρ* when *G* = 1, and is less than that of the ELS only at relatively large values of *ρ*. Similar behavior is observed under the pitching condition, except that the response of the HSLDS-FRVIS in the *z* direction is always greater than that of the ELS. However, this behavior changes when *G* = 10, where the responses of the HSLDS-FRVIS in the *z* direction are nearly always less than or equal to that of the ELS under both rolling or pitching conditions. Moreover, the gap between the two responses becomes increasingly obvious with increasing *ρ* or *φ*. This demonstrates that the displacement offset of the HSLDS-FRVIS is smaller than that of the ELS under large load and large swing angle conditions, which is beneficial for ensuring the stability of the vibration isolation system. This can be qualitatively explained from an analysis of Eqs. (5) and (7) combined with Eqs. (9) and (10) respectively, where we note that the load and swing angle have less influence on the responses of the HSLDS-FRVIS in the *z* direction than the ELS. Therefore, the HSLDS-FRVIS provides improved swing stability performance over that of the ELS.

**Figure 4:**
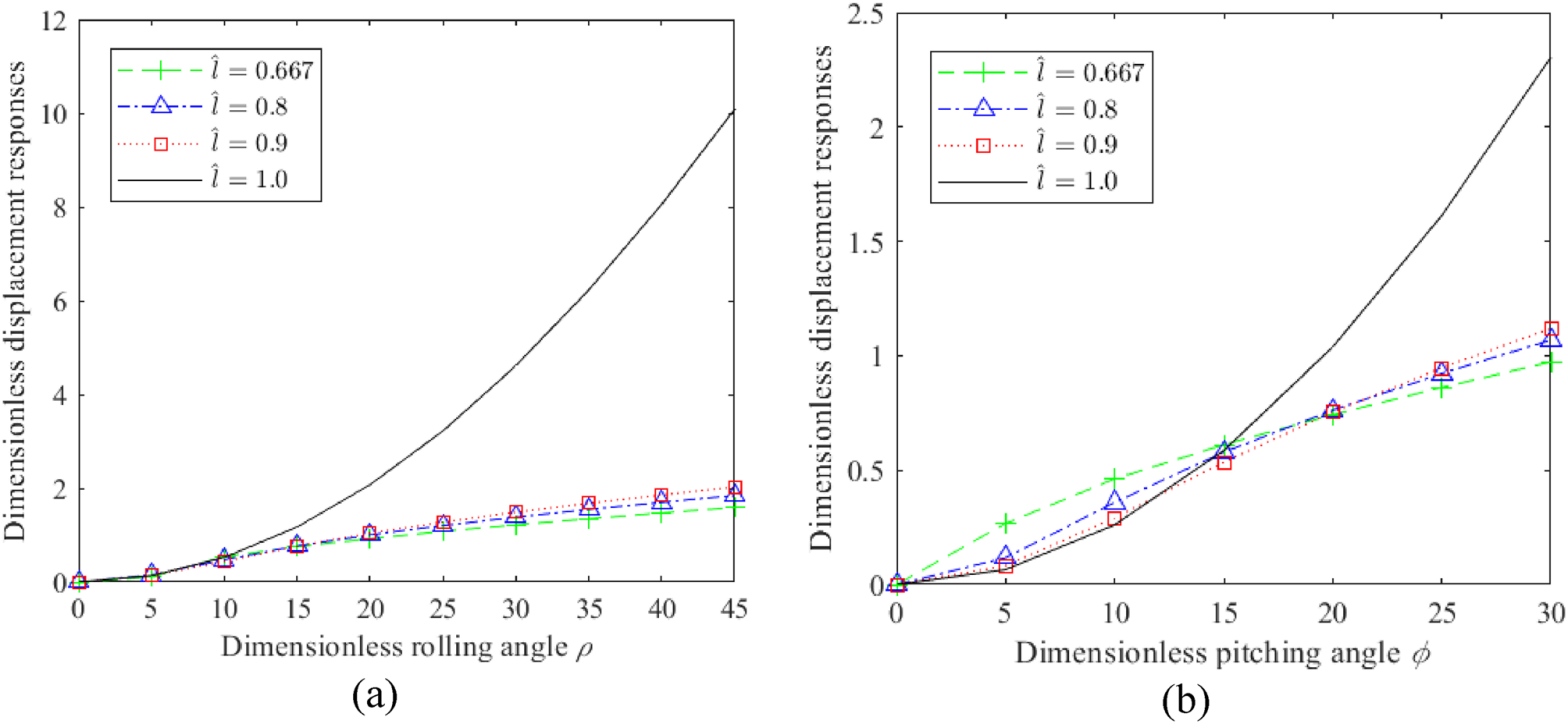
Dimensionless displacement responses obtained in the *z* direction under swing motions for the HSLDS-FRVIS and ELS as functions of *ρ* and *φ* for different degrees of nonlinearity $\hat{l}$ with $G = 10$: (**a**) rolling motion; (**b**) pitching motion.

We also analyzed the influence of the degree of nonlinearity $\hat{l}$ on the displacement responses of the HSLDS-FRVIS and the ELS in the *z* direction under swing motion for $G = 10$, and the results obtained under roll and pitch motions are presented as functions of *ρ* and *φ* in Fig. 4a and b, respectively. It is found that even slight nonlinearity can greatly reduce the displacement response of the system and improve its swing stability under heavy load. In fact, the displacement response observed for $\hat{l} = 0.9$ is little different from that obtained under the minimum displacement response at $\hat{l} = 0.667$, and is much less than that of the ELS. For example, the responses observed for $\hat{l} = 0.9$ at the maximum rolling and pitching angles considered were about 12% and 30% those of the linear system ($\hat{l} = 1.0$), respectively. Accordingly, these results reflect the high static stiffness and high static stability characteristics of HSLDS technology.

**Figure 5:**
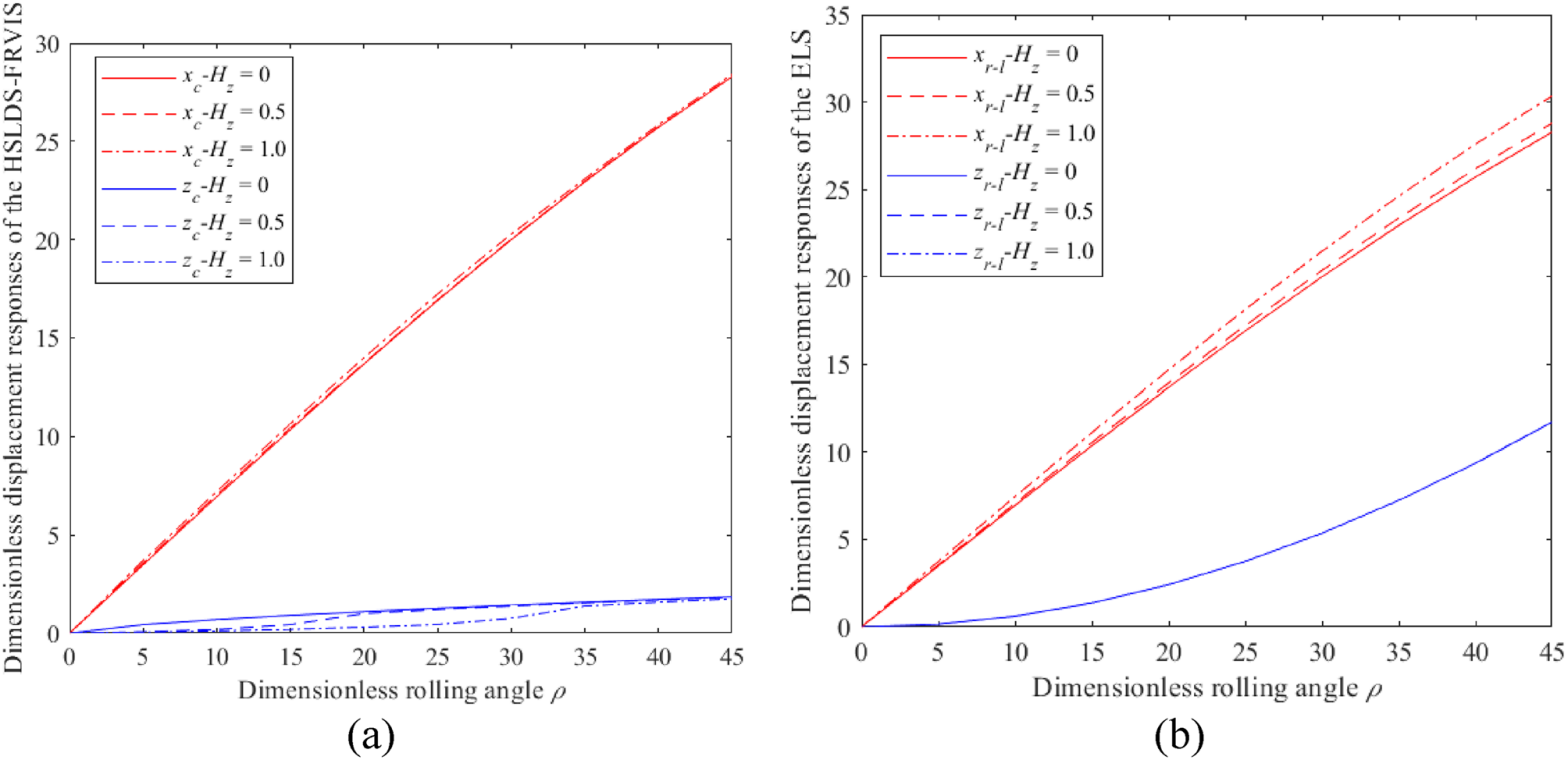
Dimensionless displacement responses obtained under rolling motion for different systems as functions of *ρ* with $G = 10$ and different vibration isolator installation positions: (**a**) HSLDS-FRVIS ($\hat{l} = 0.667$); (**b**) ELS.

Taking rolling motion as an example, we further analyzed the influence of the installation height ratio *H*_*z*_ of the vibration isolators on the displacement responses of the vibration isolation systems under rolling motion for $G = 10$, and the results obtained are presented for the HSLDS-FRVIS ($\hat{l} = 0.667$) and the ELS as functions of *ρ* in Fig. 5a and b, respectively. It is found that different installation positions have relatively little effect on the displacement response of the HSLDS-FRVIS compared to that of the ELS. We further note that *H*_*z*_ affects only the response of the ELS in the *x* direction, but has no effect on the response in the *z* direction. In addition, a value of *H*_*z*_ = 0 (i.e., *a*_*z*_ = 0) produces the minimum displacement response for the ELS in the *x* direction. This is more intuitively conveyed from an analysis of Eqs. (7) and (9). Therefore, nonlinear isolators weaken the influence of their installation position on the displacement response of the system compared with that of the ELS.

## Multidimensional dynamic analysis

Under the established condition $\xi = \xi_{x} = \xi_{y} = \xi_{z}$, the dimensionless equations defining the dynamic motion of the 6-DOF system described in “Simplified HSLDS-FRVIS model” section are given as follows.

11 $$ \left\{ \begin{aligned} & \ddot{x}_{c} + \xi \dot{x}_{c} - \xi a_{z} \dot{\beta }_{c} + k_{lx} x - k_{lx} a_{z} \beta_{c} = F_{x} \hfill \\ & \ddot{y}_{c} + \xi \dot{y}_{c} + \xi a_{z} \dot{\alpha }_{c} + k_{ly} y + k_{ly} a_{z} \alpha_{c} = F_{y} \hfill \\ & \ddot{z}_{c} + \xi \dot{z}_{c} + k_{lz} z_{c} + k_{nz} z_{c}^{3} + \frac{3}{2}k_{nz} a_{y1}^{2} z_{c} \alpha_{c}^{2} + \frac{3}{2}k_{nz} a_{y2}^{2} z_{c} \alpha_{c}^{2} + 3k_{nz} a_{x}^{2} z_{c} \beta_{c}^{2} = F_{z} \hfill \\ & \ddot{\alpha }_{c} + a_{z} k_{ly} y_{c} + a_{z} \xi \dot{y}_{c} + a_{z}^{2} k_{ly} \alpha_{c} + \frac{1}{2}a_{y1}^{2} k_{lz} \alpha_{c} + \frac{1}{2}a_{y2}^{2} k_{lz} \alpha_{c} \hfill \\ & + \frac{3}{2}a_{y1}^{2} k_{nz} z_{c}^{2} \alpha_{c} + \frac{3}{2}a_{y2}^{2} k_{nz} z_{c}^{2} \alpha_{c} + \frac{1}{2}a_{y1}^{4} k_{nz} \alpha_{c}^{3} + \frac{1}{2}a_{y2}^{4} k_{nz} \alpha_{c}^{3} \hfill \\ & + \frac{1}{2}\xi a_{y1}^{2} \dot{\alpha }_{c} + \frac{1}{2}\xi a_{y2}^{2} \dot{\alpha }_{c} + \xi a_{z}^{2} \dot{\alpha }_{c} + \frac{3}{2}a_{x}^{2} a_{y1}^{2} k_{nz} \alpha_{c} \beta_{c}^{2} + \frac{3}{2}a_{x}^{2} a_{y2}^{2} k_{nz} \alpha_{c} \beta_{c}^{2} = M_{x} \hfill \\ & \ddot{\beta }_{c} - a_{z} k_{lx} x_{c} - \xi a_{z} \dot{x}_{c} + a_{z}^{2} k_{lx} \beta_{c} + a_{x}^{2} k_{lz} \beta_{c} + 3a_{x}^{2} k_{nz} z_{c}^{2} \beta_{c} \hfill \\ & + \frac{3}{2}a_{x}^{2} a_{y1}^{2} k_{nz} \alpha_{c}^{2} \beta_{c} + \frac{3}{2}a_{x}^{2} a_{y2}^{2} k_{nz} \alpha_{c}^{2} \beta_{c} + a_{x}^{4} k_{nz} \beta_{c}^{3} + \xi a_{x}^{2} \dot{\beta }_{c} + \xi a_{z}^{2} \dot{\beta }_{c} = M_{y} \hfill \\ & \ddot{\gamma }_{c} + \frac{1}{2}a_{y1}^{2} k_{lx} \gamma_{c} + \frac{1}{2}a_{y2}^{2} k_{lx} \gamma_{c} + a_{x}^{2} k_{ly} \gamma_{c} + \xi a_{x}^{2} \dot{\gamma }_{c} + \frac{1}{2}\xi a_{y1}^{2} \dot{\gamma }_{c} + \frac{1}{2}\xi a_{y2}^{2} \dot{\gamma }_{c} = M_{z} \hfill \\ \end{aligned} \right. $$

Past research has demonstrated that little difference is observed between solutions obtained by the harmonic balance analytical method and the numerical method in the higher excitation frequency range^27^. However, these solutions differ greatly in the low frequency range because the analytical solution includes a truncation error that arises as the solution is assumed to be first order, but the actual solution has infinite order terms. Therefore, the solution obtained by the numerical method is more accurate in the low frequency range. Moreover, it is difficult to obtain explicit analytical solutions for high-dimensional nonlinear systems. Collectively considering both accuracy and simplicity, the present work applies the numerical method to solve Eq. (11) by using the ode45 solver of the MATLAB^®^ software, which also facilitates a clear and accurate analysis of the influences of various parameters on the vibration isolation performance of the system.

In addition, the present work assumes that the external excitation is simple harmonic force excitation. Therefore, the vibration isolation effect of an FRVIS is analyzed in the *x*, *y*, and *z* directions according to the force transmission rate, which is defined as

12 $$ T_{f\upsilon } = 20\lg \frac{{RMS\left( {F_{t\upsilon } } \right)}}{{RMS(F_{\upsilon } )}}({\text{dB}}) $$

Here, the function lg(∙) represents the logarithm with base 10 of its argument, the function *RMS*(∙) represents the root mean square of its argument, $F_{t\upsilon }$ is the nonlinear force transmitted by the FRVIS to the foundation, and $F_{\upsilon } = f_{\upsilon } \cos \Omega t$ is the external harmonic excitation force, where $f_{\upsilon }$ is the dimensionless amplitude of the excitation force. According to Eq. (11), the forces transmitted to the foundation in the *x*, *y*, and *z* directions can be given as follows.

13 $$ \left\{ \begin{aligned} & F_{tx} = \xi \dot{x}_{c} - \xi a_{z} \dot{\beta }_{c} + k_{lx} x - k_{lx} a_{z} \beta_{c} \hfill \\ & F_{ty} = \xi \dot{y}_{c} + \xi a_{z} \dot{\alpha }_{c} + k_{ly} y + k_{ly} a_{z} \alpha_{c} \hfill \\ & F_{tz} = \xi \dot{z}_{c} + k_{lz} z_{c} + k_{nz} z_{c}^{3} + \frac{3}{2}k_{nz} a_{y1}^{2} z_{c} \alpha_{c}^{2} + \frac{3}{2}k_{nz} a_{y2}^{2} z_{c} \alpha_{c}^{2} + 3k_{nz} a_{x}^{2} z_{c} \beta_{c}^{2} \hfill \\ \end{aligned} \right. $$

These forces can be analyzed further by considering the following two types of excitations.

1. Unidirectional excitation:
  14 $$ F_{x} = F_{y} = 0{, }F_{z} = 0.01\cos (\Omega t){, }M_{x} = M_{y} = M_{z} = 0. $$
2. Multidirectional eccentric excitation:
  15 $$ \begin{aligned} F_{x} & = F_{y} = 0.005\cos (\Omega t),F_{z} = 0.01\cos (\Omega t), \hfill \\ M_{x} & = s_{y} F_{z} - s_{z} F_{y} ,M_{y} = s_{z} F_{x} - s_{x} F_{z} ,M_{z} = s_{x} F_{y} - s_{y} F_{x} . \hfill \\ \end{aligned} $$

**Figure 6:**
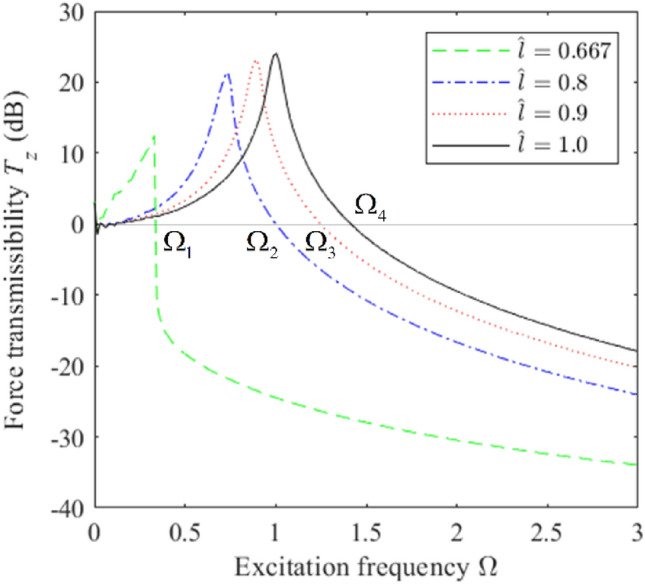
Force transmission rate *T*_*z*_ obtained in the *z* direction for an HSLDS-FRVIS as a function of the dimensionless excitation frequency Ω under various values of $\hat{l}$ with a dimensionless damping ratio $\xi = 0.06$.

As can be seen, the unidirectional excitation mode defined in Eq. (14) facilitates an analysis of the vibration isolation performance of a vibration isolation system in the *z* direction when the load is concentrated at the origin *O* of the global coordinate system (i.e., at the center of gravity of the system). We first analyze the influence of $\hat{l}$ on the *z*-directional vibration isolation performance by plotting the force transmission rate *T*_*z*_ obtained in the *z* direction as a function of Ω under various values of $\hat{l}$ with $\xi = 0.06$ in Fig. 6. The values of Ω at which *T*_*z*_ = 0 under the four values of $\hat{l}$ are given as Ω_1_–Ω_4_. It is found that the vibration isolation effect of the system in the *z* direction increases with decreasing $\hat{l}$, and achieves a maximum vibration isolation performance at $\hat{l} = 0.667$. We also considered the differences between the Ω_1_–Ω_4_ values relative to the vibration isolation frequency Ω_4_ of the linear system, and the results were ${{(\Omega_{4} - \Omega_{1} )} \mathord{\left/ {\vphantom {{(\Omega_{4} - \Omega_{1} )} {\Omega_{4} }}} \right. \kern-0pt} {\Omega_{4} }} = 76.8\%$, ${{(\Omega_{4} - \Omega_{2} )} \mathord{\left/ {\vphantom {{(\Omega_{4} - \Omega_{2} )} {\Omega_{4} }}} \right. \kern-0pt} {\Omega_{4} }} = 30.3\%$ and ${{(\Omega_{4} - \Omega_{3} )} \mathord{\left/ {\vphantom {{(\Omega_{4} - \Omega_{3} )} {\Omega_{4} }}} \right. \kern-0pt} {\Omega_{4} }} = 12.7\%$. Accordingly, an increasing degree of nonlinearity is found to expand the range of vibration isolation frequencies and improve the low-frequency vibration isolation performance of the isolator. This can be explained from an analysis of Eq. (11), where we note that the nonlinear stiffness term is zero at $\hat{l} = 1$, such that the system response is mainly composed of the dominant harmonic solution. However, the proportion of the subharmonic solution increases with decreasing $\hat{l}$ because this decreases the linear stiffness term and increases the nonlinear stiffness term, and the frequency corresponding to the peak system transmittance shifts to the left, resulting in an expanded vibration isolation frequency band.

In contrast to the above unidirectional excitation analysis, the multidirectional excitation mode defined in Eq. (15), which is common in practical engineering settings, enables the influences of nonlinearity and the installation positions of the vibration isolators on the vibration isolation performance of the HSLDS-FRVIS to be analyzed under eccentric loading at coordinates *s*_*x*_, *s*_*y*_, and *s*_*z*_. The eccentricity was set as ${{s_{x} }/{l_{c} }} = 0.8$, ${{s_{y} }/{b_{c} }} = 0.8$, and ${{s_{z} }/{h_{c} }} = - 1$.

**Figure 7:**
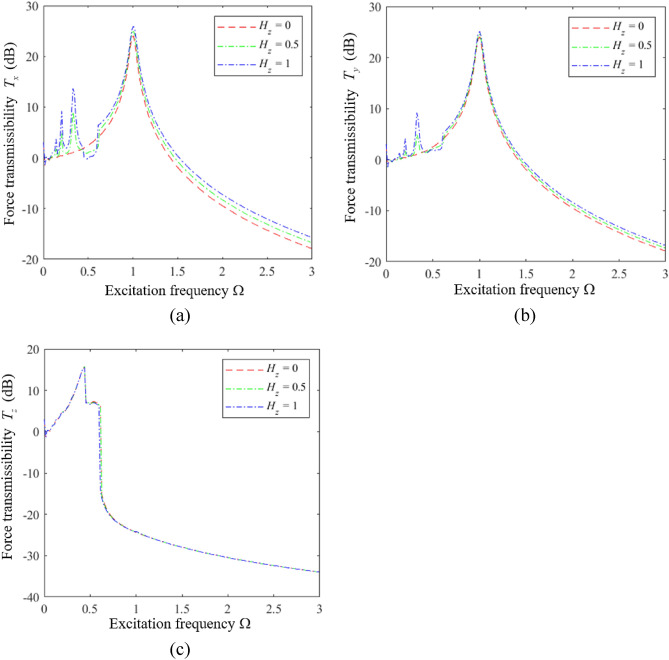
Force transmission rates *T*_*x*_, *T*_*y*_, and *T*_*z*_ obtained for an HSLDS-FRVIS in the *x*, *y*, and *z* directions, respectively, as functions of Ω under various values of *H*_*z*_ with $\hat{l} = 0.667$ and otherwise standard conditions: (**a**) *T*_*x*_; (**b**) *T*_*y*_; (**c**) *T*_*z*_.

**Figure 8:**
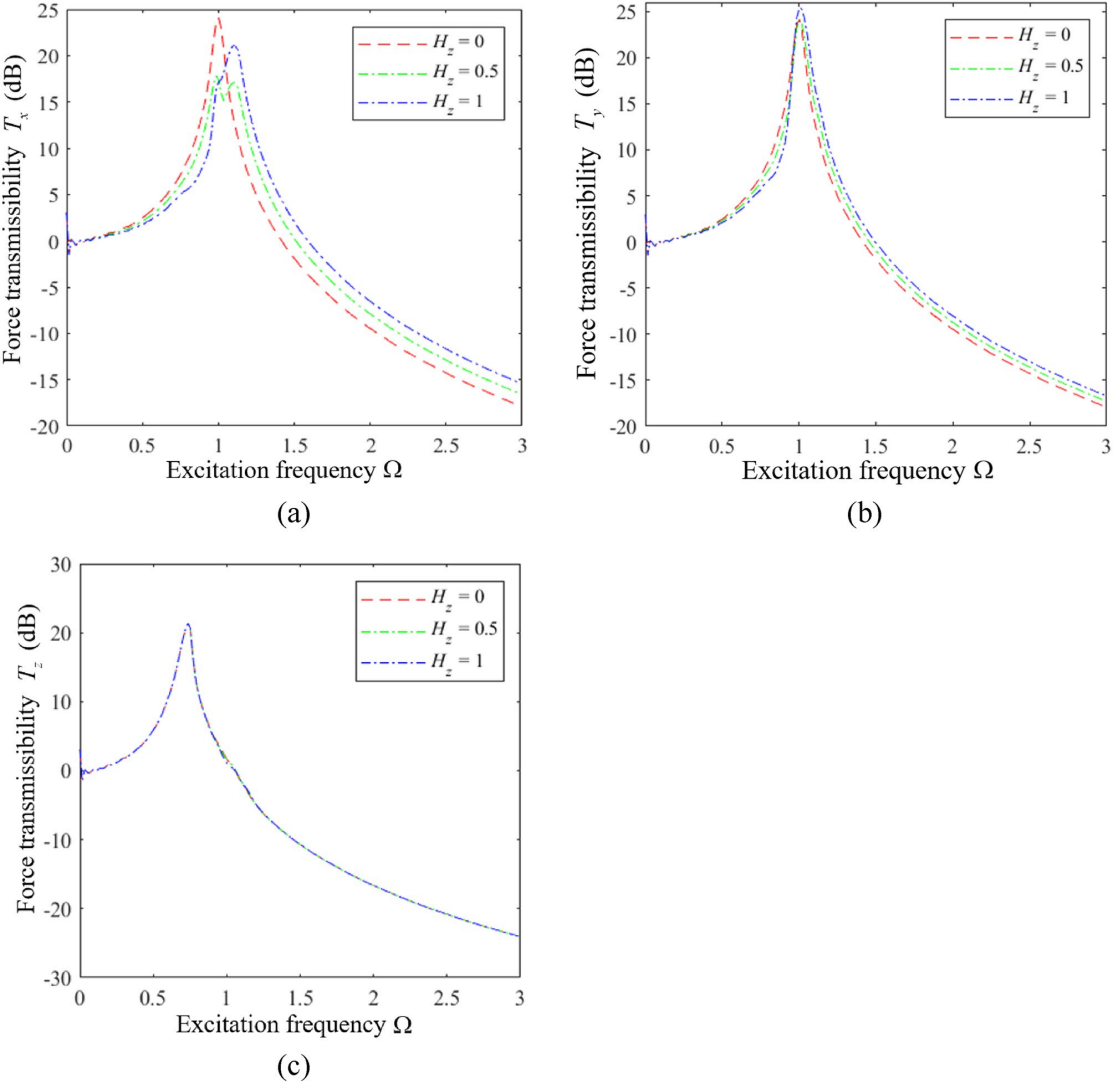
Force transmission rates obtained for an HSLDS-FRVIS as functions of Ω under various values of *H*_*z*_ with $\hat{l} = 0.8$ and otherwise standard conditions: (**a**) *T*_*x*_; (**b**) *T*_*y*_; (**c**) *T*_*z*_.

**Figure 9:**
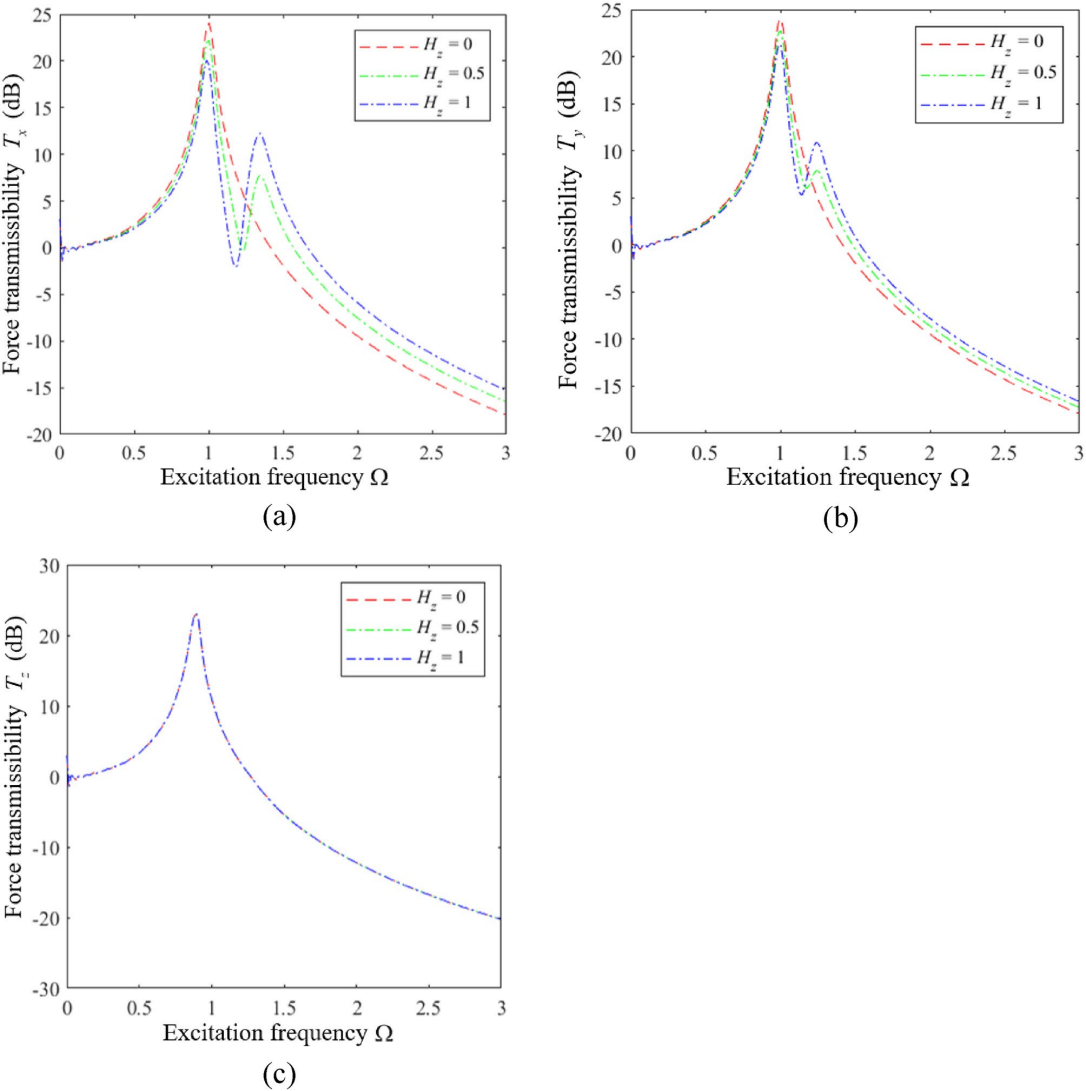
Force transmission rates obtained for an HSLDS-FRVIS as functions of Ω under various values of *H*_*z*_ with $\hat{l} = 0.9$ and otherwise standard conditions: (**a**) *T*_*x*_; (**b**) *T*_*y*_; (**c**) *T*_*z*_.

**Figure 10:**
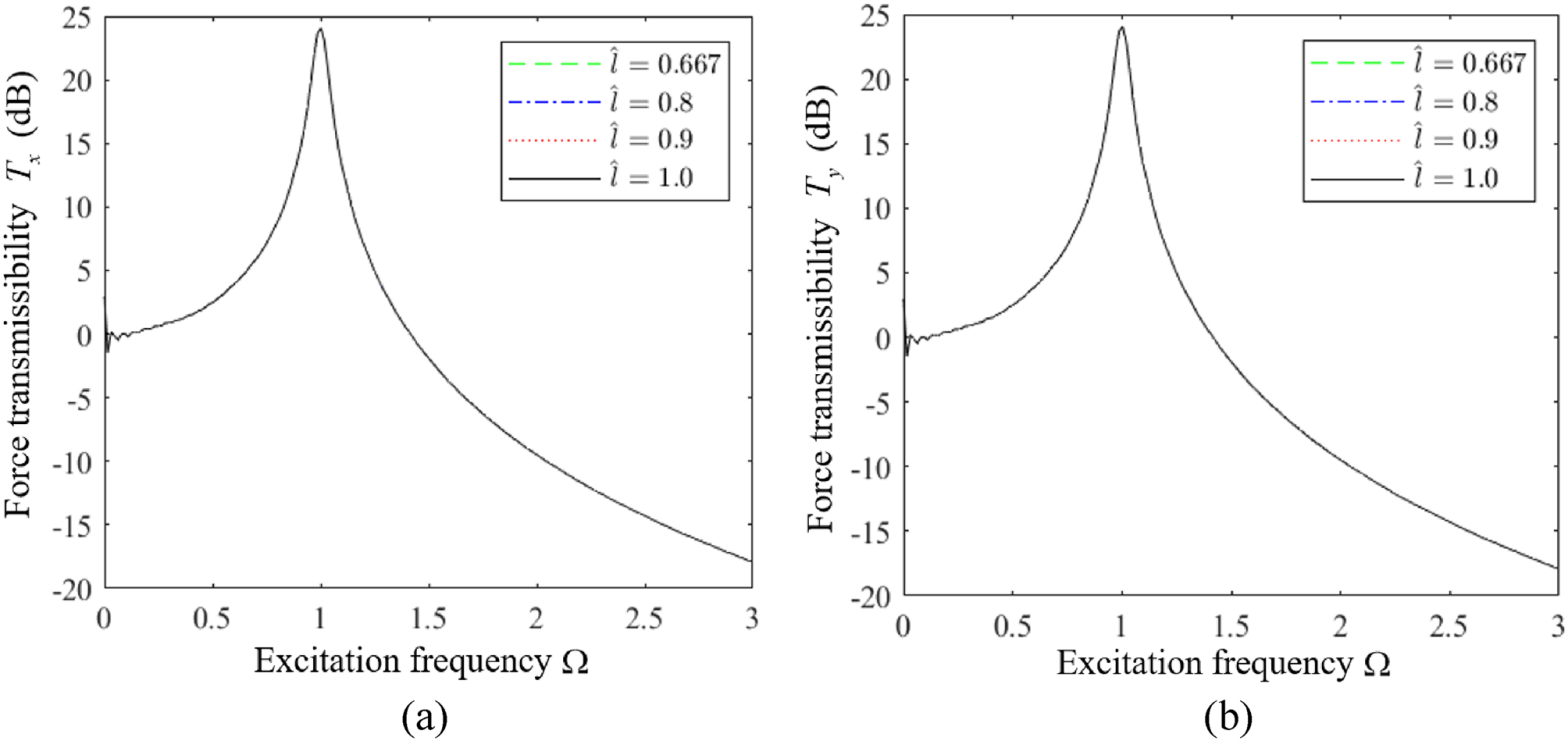
Force transmission rates obtained for an HSLDS-FRVIS as functions of Ω under various values of $\hat{l}$ with $H_{z} = 0$ and otherwise standard conditions: (**a**) *T*_*x*_; (**b**) *T*_*y*_.

We first analyze the influence of $H_{z}$ on the vibration isolation performance of an HSLDS-FRVIS with $\hat{l}$ = 0.667 and otherwise standard parameters (i.e., those in Table 1 and $\xi = 0.06$) by plotting the force transmission rates *T*_*x*_, *T*_*y*_, and *T*_*z*_ obtained in the *x*, *y*, and *z* directions in Fig. 7a–c, respectively, as functions of Ω under various values of $H_{z}$. Corresponding results are presented in Figs. 8 and 9 for $\hat{l}$ = 0.8 and $\hat{l}$ = 0.9, respectively. First of all, it is found that $H_{z}$ has little effect on the vibration isolation performance of the raft in the *z* direction, and mainly affects the isolation performance in the *x* and *y* directions. The relative impacts of $H_{z}$ in the *x*, *y*, and *z* directions can be explained from an analysis of Eq. (11), which indicates that $H_{z}$ (i.e., $a_{z}$) mainly affects the vibration response of the raft in the *x*, *y*, $\alpha$, and $\beta$ directions. Moreover, we note that the apparent impact of $H_{z}$ on the isolation performance in the *x* and *y* directions increases with increasing $\hat{l}$. In fact, the respective plots of *T*_*x*_ and *T*_*y*_ in Fig. 10a and b as functions of Ω for different values of $\hat{l}$ with $H_{z} = 0$ demonstrate that the degree of nonlinearity has no effect on the response of the raft in the *x* and *y* directions under this isolator installation condition. However, the degree of nonlinearity clearly affects the response of the raft in the *x* and *y* directions for *H*_*z*_ values of 0.5 and 1. In fact, secondary peaks are observed in the *T*_*x*_ and *T*_*y*_ spectra under these isolator installation conditions, which weakens the vibration isolation performance of the raft. Moreover, the magnitudes of these secondary peaks increase with increasing *H*_*z*_, which is an increasing detriment to the vibration isolation performance. In addition, the high-frequency vibration isolation effect of the raft in the *x* and *y* directions increases with decreasing *H*_*z*_. Therefore, an HSLDS-FRVIS design with $H_{z} = 0$ provides an overall optimal vibration isolation effect by avoiding secondary peaks in the *T*_*x*_ and *T*_*y*_ spectra and ensuring the best high-frequency vibration isolation effect in the *x* and *y* directions.

**Figure 11:**
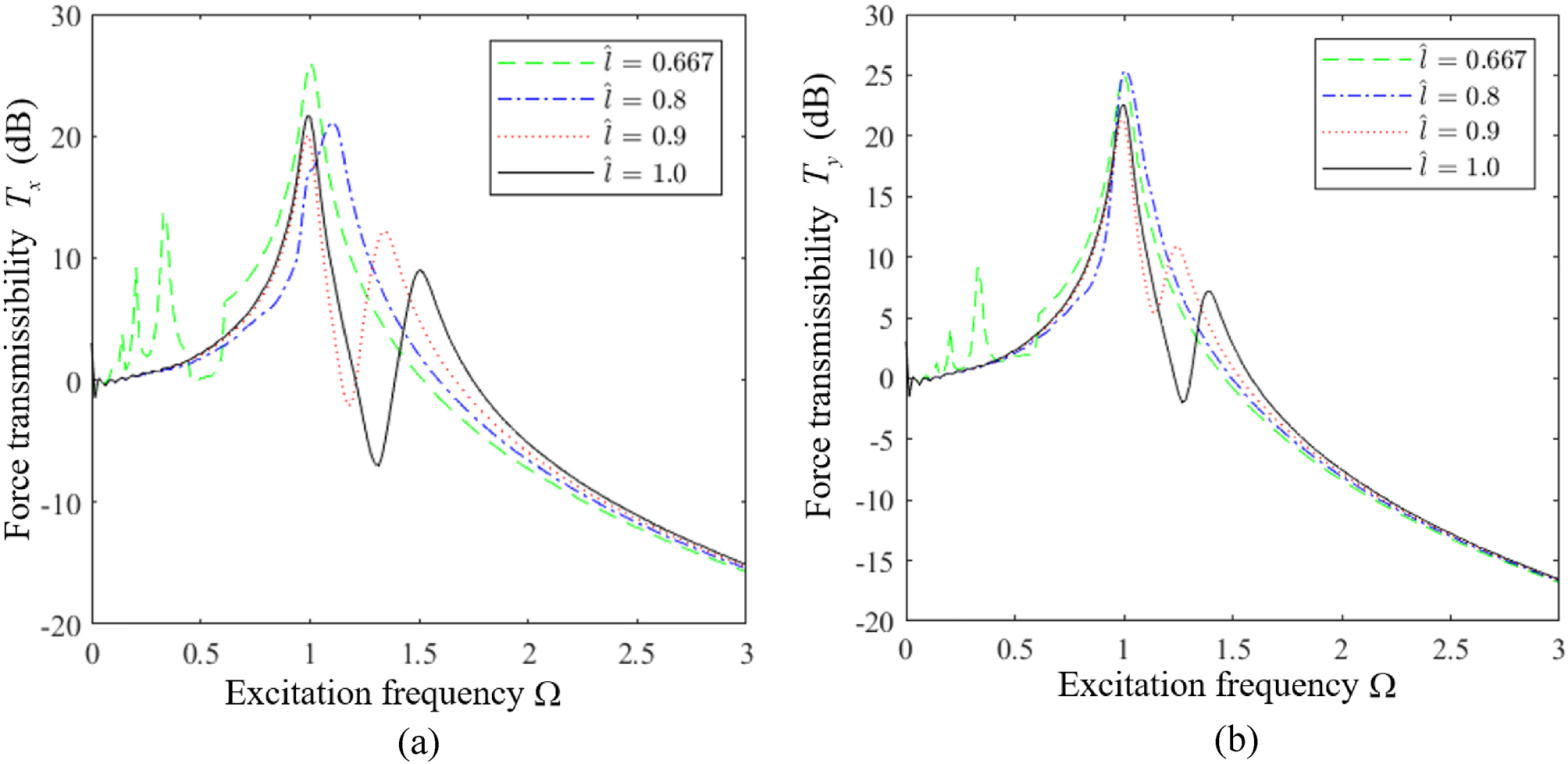
Force transmission rates obtained for an HSLDS-FRVIS as functions of Ω under various values of $\hat{l}$ with $H_{z} = 1$ and otherwise standard conditions: (**a**) *T*_*x*_; (**b**) *T*_*y*_.

However, the mechanisms by which secondary peaks arise in the *T*_*x*_ and *T*_*y*_ spectra require some analysis. This can be ascertained based on the respective plots of *T*_*x*_ and *T*_*y*_ in Fig. 11a and b as functions of Ω for different values of $\hat{l}$ with $H_{z} = 1$ as an example. We first consider the ELS ($\hat{l} = 1.0$), which yields two strong peaks in the *T*_*x*_ and *T*_*y*_ spectra. We must further note that, according to Eq. (11), the responses in the *x* and *y* directions are functions solely of a 2-DOF system. The response in the *x* direction is defined according to the following typical dynamic equations for a 2-DOF system.

16 $$ \left\{ \begin{aligned} & \ddot{x}_{c} + \xi \dot{x}_{c} - \xi a_{z} \dot{\beta }_{c} + k_{lx} x - k_{lx} a_{z} \beta_{c} = F_{x} \hfill \\ & \ddot{\beta }_{c} - a_{z} k_{lx} x_{c} - \xi a_{z} \dot{x}_{c} + a_{z}^{2} k_{lx} \beta_{c} + a_{x}^{2} k_{lz} \beta_{c} + \xi a_{x}^{2} \dot{\beta }_{c} + \xi a_{z}^{2} \dot{\beta }_{c} = M_{y} \hfill \\ \end{aligned} \right. $$

The *y*-direction responses are the same. Therefore, the system generates two transmission peaks in both the *x* and *y* directions. We further note that the strongly nonlinear QZS system ($\hat{l} = 0.667$) generates a large number of secondary spectra at frequencies less than the primary peak, which detracts from the low-frequency vibration isolation effect of the raft. These secondary spectra arise because the linear stiffness term in the *z* direction is zero when $\hat{l} = 0.667$, and the strong nonlinearity in the *z* direction leads to subharmonic solutions with large magnitudes in the *x* and *y* directions. As can be observed, these secondary peaks do not appear for weakly nonlinear systems ($\hat{l} = 0.8$ and $\hat{l} = 0.9$), which either exhibit responses that represent a combination of the responses of the ELS and the QZS system for $\hat{l} = 0.8$, or responses that are quite similar to the ELS for $\hat{l} = 0.9$. This is because the nonlinear stiffness term of the system decreases with increasing $\hat{l}$, while the linear stiffness term increases, and this reduces the magnitude of the subharmonic responses obtained in the solution.

**Figure 12:**
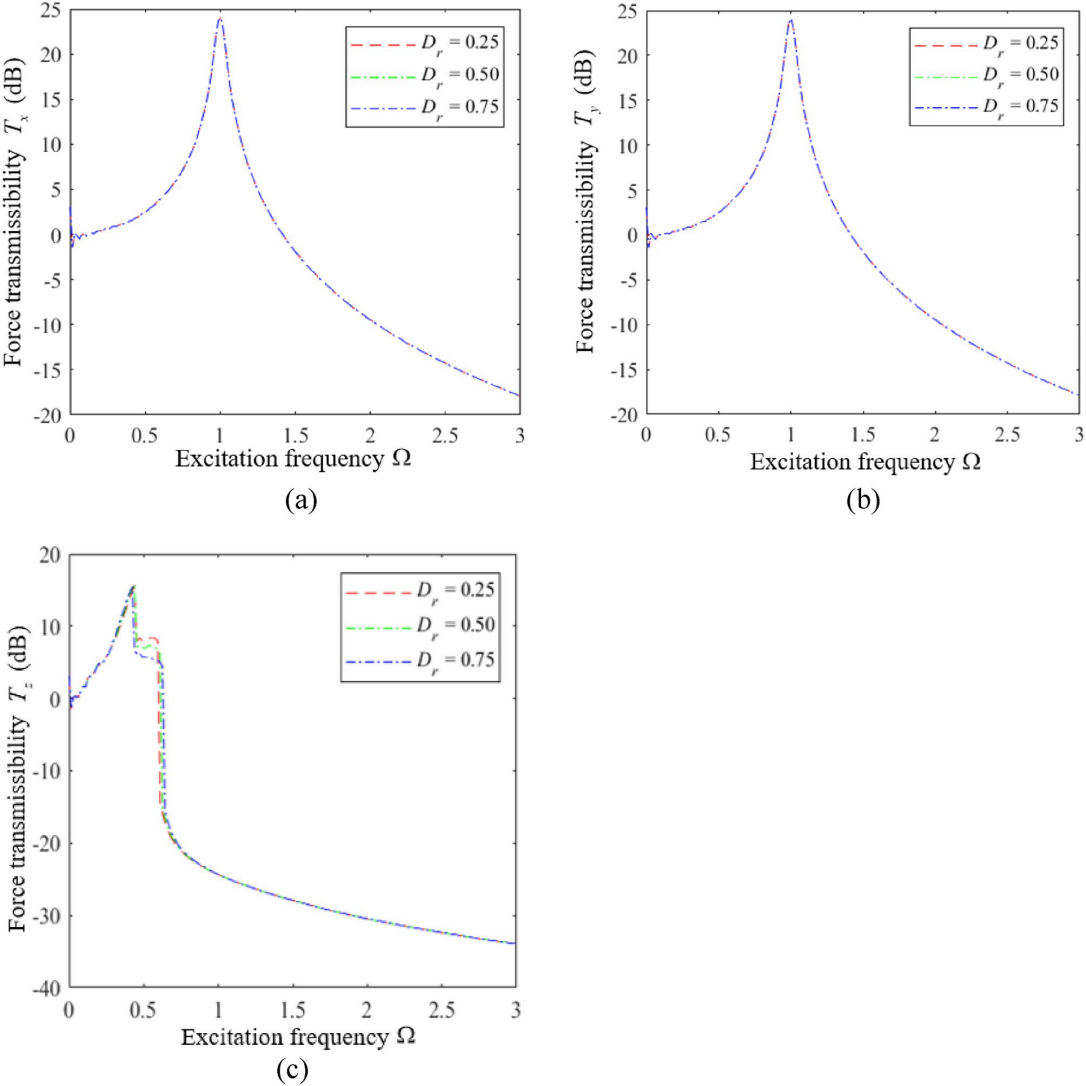
Force transmission rates obtained for an HSLDS-FRVIS as functions of Ω under various values of *D*_*r*_ with $\hat{l} = 0.667$ and otherwise standard conditions: (**a**) *T*_*x*_; (**b**) *T*_*y*_; (**c**) *T*_*z*_.

**Figure 13:**
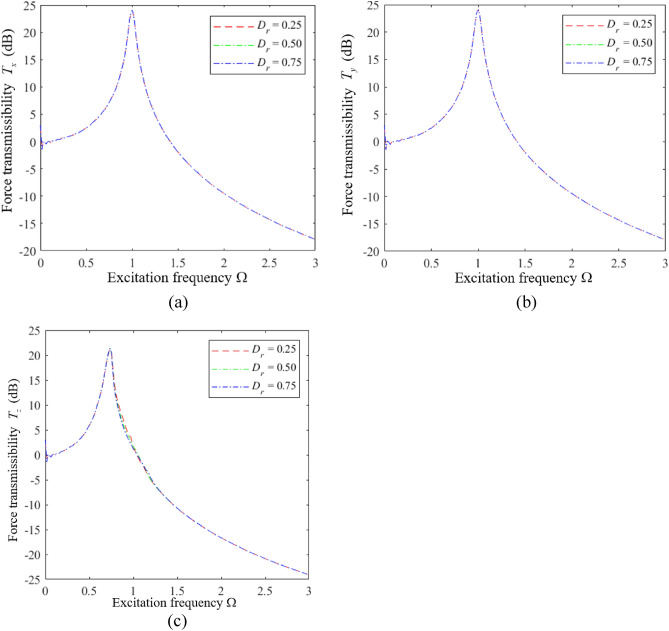
Force transmission rates obtained for an HSLDS-FRVIS as functions of Ω under various values of *D*_*r*_ with $\hat{l} = 0.8$ and otherwise standard conditions: (**a**) *T*_*x*_; (**b**) *T*_*y*_; (**c**) *T*_*z*_.

Finally, the influence of the distance ratio $D_{r}$ on the vibration isolation performance of the HSLDS-FRVIS was analyzed according to the plots of *T*_*x*_, *T*_*y*_, and *T*_*z*_ presented as functions of Ω under various values of $D_{r}$ with $\hat{l} = 0.667$ in Fig. 12a–c, respectively. Corresponding results are presented in Fig. 13 for $\hat{l}$ = 0.8. It can be found that $D_{r}$ has little effect on the vibration isolation performance of the HSLDS-FRVIS in any direction. Therefore, an equidistant installation with $D_{r} = 0.5$ would be considered ideal from the standpoint of installation stability.

## Conclusion

The present work addressed the poorly developed process for designing the complex high-dimensional HSLDS-FRVISs applied in ocean-going vessels by establishing a 6-DOF HSLDS-FRVIS model, and applying that model for fully analyzing the swing stability and multidimensional vibration isolation performance of these systems. The results of extensive analysis demonstrate the following conclusions.

1. Weak nonlinearity greatly reduces the swing displacement responses of the system and improves the swing stability under heavy loads and large swing angles. This reflects the high static stiffness and high static stability characteristics of HSLDS systems. In addition, applying different installation positions for the vibration isolators has little effect on the displacement responses of the HSLDS systems in comparison with that of the corresponding linear systems. Meanwhile, the influence of the installation position on the swing displacement responses of the system decreases with increasing nonlinearity.
2. In terms of the multidimensional vibration isolation performance of the systems, the low-frequency vibration isolation effect can increase with increasing nonlinearity. However, a strongly nonlinear QZS system is more sensitive to parameter changes than weakly nonlinear HSLDS systems. The results of analysis demonstrate that applying a value of *H*_*z*_ = 0 produces the best vibration isolation performance overall under strong nonlinearity by avoiding unnecessary secondary peaks in the force transmission rate under harmonic mechanical excitation and ensuring a maximum high-frequency vibration isolation effect. However, applying a weak nonlinearity is better than a strong nonlinearity if *H*_*z*_ is not zero. Therefore, a weakly nonlinear HSLDS system is preferred to a highly nonlinear QZS system in engineering design practice to ensure an optimal vibration isolation performance. Meanwhile, $D_{r}$ has little impact on the vibration isolation effects of the system in *x*, *y*, and *z* directions. Therefore, applying an equidistant installation with $D_{r} = 0.5$ would be considered ideal from the standpoint of installation stability.

Accordingly, the current work lays a sound theoretical foundation for the subsequent design of HSLDS-FRVISs.

## Data Availability

Some of the data and models generated during the study are available from the corresponding author by reasonable request.

## References

[1] Howard, C. Q. Recent developments in submarine vibration isolation and noise control. In *Proceedings of 1st Submarine Science Technology and Engineering Conference, Adelaide, SA, Australia* (2011).

[2] Vane, F. *A Guide for the Selection and Application of Resilient Mountings to Shipboard Equipment (Revised)*. David Taylor Model Basin Report 880 (1958).

[3] Carrella, A., Brennan, M. J. & Waters, T. P. Static analysis of a passive vibration isolator with quasi-zero-stiffness characteristic. *J. Sound Vib.* **301**, 678–689 (2007). 10.1016/j.jsv.2006.10.011

[4] Carrella, A., Brennan, M. J., Waters, T. P. & Shin, K. On the design of a high-static–low-dynamic stiffness isolator using linear mechanical springs and magnets. *J. Sound Vib.* **315**, 712–720 (2008). 10.1016/j.jsv.2008.01.046

[5] Alabuzhev, P. & Rivin, E. *Vibration Protecting and Measuring Systems with Quasi-Zero Stiffness* (Hemisphere Publishing Corporation, 1989).

[6] Gatti, G., Shaw, A. D., Gonçalves, P. J. P. & Brennan, M. J. On the detailed design of a quasi-zero stiffness device to assist in the realisation of a translational Lanchester damper. *Mech. Syst. Signal Process.* **164**, 108258 (2022). 10.1016/j.ymssp.2021.108258

[7] Shaw, A. D., Gatti, G., Gonçalves, P. J. P., Tang, B. & Brennan, M. J. Design and test of an adjustable quasi-zero stiffness device and its use to suspend masses on a multi-modal structure. *Mech. Syst. Signal Process.* **152**, 107354 (2021). 10.1016/j.ymssp.2020.107354

[8] Ishida, S., Uchida, H., Shimosaka, H. & Hagiwara, I. Design and numerical analysis of vibration isolators with quasi-zero-stiffness characteristics using bistable foldable structures. *J. Vib. Acoust.* **139**, 031015 (2017). 10.1115/1.4036096

[9] Ishida, S., Suzuki, K. & Shimosaka, H. Design and experimental analysis of origami-inspired vibration isolator with quasi-zero-stiffness characteristic. *J. Vib. Acoust.* **139**, 051004–051012 (2017). 10.1115/1.4036465

[10] Xu, D., Zhang, Y., Zhou, J. & Lou, J. On the analytical and experimental assessment of the performance of a quasi-zero-stiffness isolator. *J. Vib. Control* **20**, 2314–2325 (2014). 10.1177/1077546313484049

[11] Zhang, Y., Wei, G., Wen, H., Jin, D. & Hu, H. Design and analysis of a vibration isolation system with cam–roller–spring–rod mechanism. *J. Vib. Control* **28**, 1781–1791 (2022). 10.1177/10775463211000516

[12] Yao, Y., Wang, X. & Li, H. Design and analysis of a high-static-low-dynamic stiffness isolator using the cam-roller-spring mechanism. *J. Vib. Acoust.* **142**, 021009 (2020). 10.1115/1.4045583

[13] Han, J., Meng, L. & Sun, J. Design and characteristics analysis of a nonlinear isolator using a curved-mount-spring-roller mechanism as negative stiffness element. *Math. Probl. Eng.* **2018**, 1359461 (2018).

[14] Wang, K., Zhou, J. & Xu, D. Sensitivity analysis of parametric errors on the performance of a torsion quasi-zero-stiffness vibration isolator. *Int. J. Mech. Sci.* **134**, 336–346 (2017). 10.1016/j.ijmecsci.2017.10.026

[15] Zhou, J., Xu, D. & Bishop, S. A torsion quasi-zero stiffness vibration isolator. *J. Sound Vib.* **338**, 121–133 (2015). 10.1016/j.jsv.2014.10.027

[16] Vo, N. Y. P. & Le, T. D. Dynamic analysis of quasi-zero stiffness pneumatic vibration isolator. *Appl. Sci.* **12**, 2378 (2022). 10.3390/app12052378

[17] Vo, N. Y. P., Nguyen, M. K. & Le, T. D. Analytical study of a pneumatic vibration isolation platform featuring adjustable stiffness. *Commun. Nonlinear Sci. Numer. Simul.* **98**, 105775 (2021). 10.1016/j.cnsns.2021.105775

[18] Shuai, C., Li, B. & Ma, J. A novel multi-directional vibration isolation system with high-static–low-dynamic stiffness. *Acta Mech.* **233**, 5199–5214 (2022). 10.1007/s00707-022-03387-0

[19] Zhou, Z., Zhou, M., Dai, Z., Liu, X. & Li, Z. Design and experimental validation of a vibration isolator with high-static low-dynamic stiffness and operating point variable property. *J. Vib. Control* **28**, 1341–1350 (2022). 10.1177/1077546321990524

[20] Jiang, Y., Song, C., Ding, C. & Xu, B. Design of magnetic-air hybrid quasi-zero stiffness vibration isolation system. *J. Sound Vib.* **477**, 115346 (2020). 10.1016/j.jsv.2020.115346

[21] Yuan, S. *et al.* Tunable negative stiffness spring using Maxwell normal stress. *Int. J. Mech. Sci.* **193**, 106127 (2021). 10.1016/j.ijmecsci.2020.106127

[22] Yuan, S. *et al.* A tunable quasi-zero stiffness isolator based on a linear electromagnetic spring. *J. Sound Vib.* **482**, 115449 (2020). 10.1016/j.jsv.2020.115449

[23] Sun, B. & Jing, X. A tracked robot with novel bio-inspired passive “legs”. *Robot. Biomim.* **4**, 18 (2017). 10.1186/s40638-017-0070-6 PMC5684273 29201601

[24] Jiang, G., Jing, X. & Guo, Y. A novel bio-inspired multi-joint anti-vibration structure and its nonlinear HSLDS properties. *Mech. Syst. Signal Process.* **138**, 106552 (2020). 10.1016/j.ymssp.2019.106552

[25] Jing, X., Zhang, L., Feng, X., Sun, B. & Li, Q. A novel bio-inspired anti-vibration structure for operating hand-held jackhammers. *Mech. Syst. Signal Process.* **118**, 317–339 (2019). 10.1016/j.ymssp.2018.09.004

[26] Dai, H., Jing, X., Wang, Y., Yue, X. & Yuan, J. Post-capture vibration suppression of spacecraft via a bio-inspired isolation system. *Mech. Syst. Signal Process.* **105**, 214–240 (2018). 10.1016/j.ymssp.2017.12.015

[27] Li, Y. & Xu, D. Vibration attenuation of high dimensional quasi-zero stiffness floating raft system. *Int. J. Mech. Sci.* **126**, 186–195 (2017). 10.1016/j.ijmecsci.2017.03.029

[28] Li, Y. & Xu, D. Force transmissibility of floating raft systems with quasi-zero-stiffness isolators. *J. Vib. Control* **24**, 3608–3616 (2018). 10.1177/1077546317708460

[29] He, L., Xu, W., Bu, W. & Shi, L. Dynamic analysis and design of air spring mounting system for marine propulsion system. *J. Sound Vib.* **333**, 4912–4929 (2014). 10.1016/j.jsv.2014.05.045

[30] Xu, X., Liu, H., Jiang, X. & Atindana, A. V. Uncertainty analysis and optimization of quasi-zero stiffness air suspension based on polynomial chaos method. *Chin. J. Mech. Eng.* **35**, 93 (2022). 10.1186/s10033-022-00758-5

[31] Wang, X., Liu, H., Chen, Y. & Gao, P. Beneficial stiffness design of a high-static-low-dynamic-stiffness vibration isolator based on static and dynamic analysis. *Int. J. Mech. Sci.* **142–143**, 235–244 (2018). 10.1016/j.ijmecsci.2018.04.053

[32] Lu, Z., Chen, L., Brennan, M. J., Li, J.-M. & Ding, H. The characteristics of vibration isolation system with damping and stiffness geometrically nonlinear. *J. Phys. Conf. Ser.* **744**, 012115 (2016). 10.1088/1742-6596/744/1/012115

[33] *Harris’ Shock and Vibration Handbook*. (McGraw-Hill, 2010).

[34] Tang, B. & Brennan, M. J. On the shock performance of a nonlinear vibration isolator with high-static-low-dynamic-stiffness. *Int. J. Mech. Sci.* **81**, 207–214 (2014). 10.1016/j.ijmecsci.2014.02.019

[35] Carrella, A., Brennan, M. J., Waters, T. P. & Lopes, V. Force and displacement transmissibility of a nonlinear isolator with high-static-low-dynamic-stiffness. *Int. J. Mech. Sci.* **55**, 22–29 (2012). 10.1016/j.ijmecsci.2011.11.012

